# Low-intensity versus moderate- to high-intensity lipid-lowering therapy after myocardial infarction in patients aged 80 years and older: A retrospective cohort study

**DOI:** 10.1016/j.ajpc.2025.101391

**Published:** 2025-12-21

**Authors:** Shichen Jiang, Linjie Li, Yuyang Miao, Geru Aa, Hangkuan Liu, Xiaozhi Chen, Haonan Sun, Yiwen Fang, Pengfei Sun, Xin Zhou, Qiang Zhang

**Affiliations:** a Department of Cardiology, Tianjin Medical University General Hospital, 154 Anshan Road, Heping District, Tianjin 300052, China; b Department of Geriatrics, Tianjin Medical University General Hospital, Key Laboratory of Post-Neuro injury and Regeneration in Central Nervous System, Ministry of Education, State Key Laboratory of Experimental Hematology, Tianjin Key Laboratory of Elderly Health, Tianjin Geriatrics Institute, Tianjin, China

**Keywords:** Elderly, Myocardial infarction, Lipid-lowering therapy, Mortality

## Abstract

**Background:**

Current evidence regarding the optimal intensity of lipid-lowering therapy (LLT) for post-myocardial infarction (MI) patients aged over 80 years remains insufficient.

**Methods:**

This analysis was performed in patients aged over 80 years following MI, using data from the Tianjin Health and Medical Data Platform. The exposure was intensity of LLT (low-intensity versus moderate- to high-intensity). The primary outcome was all-cause mortality, with secondary outcomes including cardiovascular mortality, recurrent MI and stroke. Multivariable-adjusted Cox model was used to calculated hazard ratio (HR) and 95 % confidence interval (CI). Charlson Comorbidity Index (CCI) score was used to stratify the cohort.

**Results:**

Among the 11,585 patients, 3559 received low-intensity LLT, and 8026 received moderate- to high-intensity LLT, with mortality rates of 29.1 % and 21.4 % respectively during a median follow-up of 3 years. Compared with low-intensity LLT group, moderate- to high-intensity LLT was associated with a statistically significant reduction in all-cause mortality (HR, 0.81 [95 % CI: 0.75–0.88]) and cardiovascular mortality (HR, 0.77 [95 % CI: 0.70–0.86]). The multivariable fractional polynomial interaction analysis revealed that only patients with CCI scores ≤ 4 derived significantly both all-cause mortality and cardiovascular mortality reduction from moderate-to-high-intensity LLT (HR, 0.79 [95 % CI: 0.72–0.87]; HR, 0.74 [95 % CI: 0.65–0.84], respectively).

**Conclusion:**

Among MI patients aged over 80 years, moderate-to-high-intensity LLT significantly reduced mortality risk during 3-year follow-up compared to low-intensity LLT only in patients with CCI scores ≤ 4. Further investigation is required to optimize personalized lipid management through rigorous assessment of LLT benefits versus adverse effects.

## Graphical abstract

**Figure:**
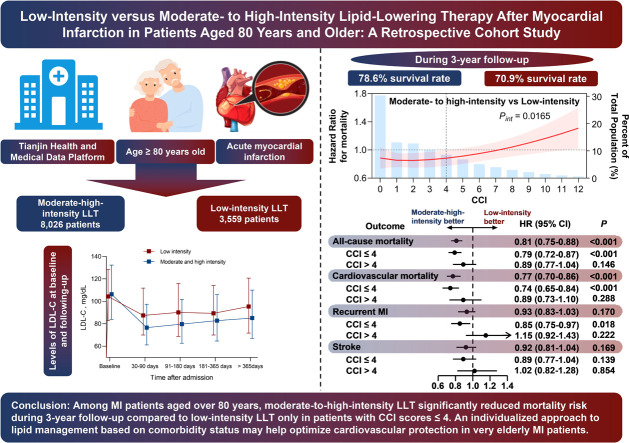

## 1 Introduction

Atherosclerotic cardiovascular diseases are the leading causes of mortality worldwide and impose a substantial health burden, particularly in aging populations [1]. Low-density lipoprotein cholesterol (LDL-C) is a well-established risk factor for atherosclerotic cardiovascular diseases [2], and lipid-lowering therapies (LLT), primarily statins, have demonstrated significant efficacy in reducing fatal and nonfatal cardiovascular events across diverse patient populations after myocardial infarction (MI) [[3], [4], [5], [6], [7]]. However, current randomized controlled trials have not specifically focused on truly elderly patients (i.e., aged over 80 years) with MI, consequently there remains a paucity of robust evidence regarding whether this vulnerable population could derive clinical benefits from moderate- to high-intensity LLT. Despite the focus on the advantages of LLT in older adults, the PROSPER trial and the meta-analysis by Gencer B et al. included participants 70 to 82 years of age (mean age, 75 years) and those 75 years of age or older (mean age, 79 years), respectively [[8], [9], [10]].

Meanwhile, the prevalence of multimorbidity, defined as the occurrence of two or more chronic conditions, is rising throughout the globe, especially among the elderly [11,12]. Decision making in LLT is complicated by multiple factors including time to benefit, limited life expectancy, competing risks of mortality, concerns regarding adverse effects, drug-drug interactions, and common geriatric syndromes such as frailty and cognitive impairment [[13], [14], [15]]. As a result, personalized LLT is imperative for older adults with MI. However, current randomized trials or guideline remains insufficient to guide comorbidity-stratified treatment in this high-risk, understudied population [[16], [17], [18]].

To address current research gaps, this study aims to evaluate, based on real-world data in patients over 80 years old with MI: 1) the association between different intensities of LLT and long-term clinical outcomes; 2) the impact of comorbidity burden on the effectiveness of LLT.

## 2 Methods

### 2.1 Data sources

The data for this study were obtained from an integrated repository encompassing comprehensive healthcare records from multiple medical centres in China (details in **Supplement Material** or our published research[[19], [20], [21]]). This investigation received formal authorization from local health commission and was granted ethical approval by the institutional review board of authors’ affiliation (IRB2024-YX-131–01). The requirement for informed consent was waived pursuant to the retrospective observational design. This study followed the Strengthening the Reporting of Observational Studies in Epidemiology reporting guideline.

### 2.2 Study population

This study included patients hospitalized with MI aged over 80 years from January 1, 2013, to December 31, 2022. MI was identified using discharge diagnosis codes I21 and I22.

The exclusion criteria included the following: patients with missing hospitalization information and without LLT during hospitalization. Considering that early mortality in MI patients is strongly associated with the initial event, and clinicians tend to prescribe more intensive LLT for “more healthy” patients [22], we excluded patients who died within 30 days of admission to reduce selection bias.

### 2.3 LLT intensity and outcomes

LLT was defined as the use of statins, ezetimibe, or proprotein convertase subtilisin/kexin type 9 (PCSK9) inhibitor during hospitalization. Low-intensity LLT was defined as 5 mg rosuvastatin, 10 mg and 15 mg atorvastatin, or any other statins every day. Moderate- to high-intensity LLT was defined as 10 mg or more rosuvastatin, 20 mg or more atorvastatin, PCSK9 inhibitor, or any combination of ezetimibe, PCSK9 inhibitor and statins [[23], [24], [25]]. The participants were grouped based on the last prescription during hospitalization.

The primary outcome was all-cause mortality within three years from admission. The secondary outcomes included cardiovascular mortality, recurrent MI, and stroke. The information was obtained from death registry in community healthcare centres. These mortality data were regularly updated and further validated through review of inpatient and outpatient medical records. The ICD codes used to identify these events are provided in **Supplemental Table 1**.

### 2.4 Covariates

The study variables included demographics (age, sex, marital status), type of MI [ST-elevation myocardial infarction (STEMI), non-ST-elevation myocardial infarction], medical history [old MI, history of percutaneous coronary intervention (PCI), diabetes mellitus, hypertension, heart failure, atrial fibrillation, chronic kidney diseases, ischaemic stroke, haemorrhagic stroke, chronic obstructive pulmonary disease, cancer and peripheral vascular disease], laboratory tests [serum levels of total cholesterol, LDL-C, high density lipoprotein cholesterol (HDL-C), triglycerides, estimated glomerular filtration rate (eGFR), haemoglobin and platelet], pre-hospital medications in the past 6 months [antiplatelet drugs (aspirin or P2Y12 inhibitors), statins, oral anticoagulants, *β*-blockers, angiotensin converting enzyme inhibitors (ACEI)/angiotensin receptor blockers (ARB), calcium channel blockers, diuretic and antidiabetic drug], and in-hospital treatments [dual antiplatelet therapy (DAPT) (aspirin and P2Y12 inhibitors), anticoagulation therapy, ACEI/ARB, *β*-blockers, calcium channel blockers, diuretic, antidiabetic drug, proton pump inhibitor and PCI]. The definitions of the above study variables are shown in the **Supplemental Table 2**.

According to patient diagnostic information, we calculated a comorbidity score for each patient using the Charlson Comorbidity Index (CCI) [26]. The CCI scoring system comprises 19 disease conditions, including MI, congestive heart failure, peripheral vascular disease, cerebrovascular disease, dementia, chronic pulmonary disease, rheumatic disease, peptic ulcer disease, mild liver disease, diabetes without chronic complications, moderate to severe renal disease, diabetes with chronic complications, hemiplegia or paraplegia, nonmetastatic solid tumor, moderate to severe liver disease, leukemia, lymphoma, metastatic solid tumour, and acquired immune deficiency syndrome.

### 2.5 Statistical analyses

Continuous variables were reported as medians (25th-75th percentile). Categorical variables were presented as percentages. Groups were compared by Kruskal-Wallis test for continuous variables and chi-square or Fisher exact tests for discrete variables. The missing baseline variables were imputed by *MissForest*, a random forest imputation algorithm for missing data implemented in R. The proportion of missing baseline variables before imputing was listed in **Supplemental Table 3**.

To evaluate the effects of LLT, we calculated LDL-C concentrations for both treatment cohorts from 30 days to 3 years post-admission. These measurements were stratified into four distinct temporal intervals: 30–90 days, 91–180 days, 181–365 days, and >365 days. Cumulative mortality risk, stratified by the intensity of LLT, was constructed using the Kaplan-Meier method. Cox regression models were used to calculate hazard ratios (HR) and 95 % confidence intervals (CI). The study utilized multivariable adjusted Cox model and covariates were as described earlier. In addition, the study also utilized propensity-score matching (PSM) analyses with a maximum 1-to-1 matching to evaluate the association between intensity of LLT and mortality. PSM was conducted using the "calipmath" command in Stata, applying a greedy matching algorithm without replacement. A caliper width of 0.2 times the standard deviation of the log-transformed propensity score was used for all matches.

We conducted subgroup analyses and interaction tests to evaluate the effects of LLT on long-term mortality, including the following variables: age, sex, type of MI, heart failure, atrial fibrillation, LDL-C levels at baseline (< 100 or ≥ 100 mg/dL), eGFR at baseline (< 60 or ≥ 60 mL/min/1.73 m^2^), pre-hospital statins in the past 6 months and in-hospital therapy (DAPT, ACEI/ARB, *β*-blockers and PCI). We compared characteristics within subgroups that exhibited significant effect differences in the subgroup analysis.

To identify the appropriate population for moderate- to high-intensity LLT, we calculated each patient’s comorbidity score using the CCI, [26] and applied the multivariable fractional polynomial interaction (MFPI) to explore potential interactions between comorbidity score and the intensity of LLT concerning outcomes. Kaplan-Meier curves were constructed for the low-intensity LLT and moderate- to high-intensity LLT groups, stratified by different CCI scores, and compared using the log-rank test. Cox regression models were used to calculate HR and 95 % CI.

In the aforementioned exclusion criteria, we excluded patients with missing baseline LDL-C, a key variable. To validate the robustness of our findings, we retained these patients and repeated the primary analysis in sensitivity analyses: 1) after imputing missing baseline LDL-C values by *MissForest*, and 2) without adjusting for baseline LDL-C. In addition, given the competing risk, we validated the results using the Fine-Gray proportional subdistribution hazards model. Specifically, for the outcome of cardiovascular mortality, we accounted for competing risk of non-cardiovascular mortality; for recurrent MI and stroke, all-cause mortality was defined as the competing event.

The Stata (version16.0, StataCorp, College Station, TX, USA) and R (version 4.1.3) was used to perform analysis. A two tailed *P* < 0.05 was considered to indicate statistical significance.

## 3 Results

### 3.1 Patients characteristics

A total of 23,478 patients aged over 80 years who experienced MI were identified. Among them, 7592 were excluded due to missing data: 6872 had missing baseline serum LDL-C, 974 had unreasonably recorded statin dosage, and 7 lacked information of time of death. Additionally, 2556 patients had no recorded use of LLT during their hospital stay, and 1438 patients who died within 30 days of admission were also excluded. Ultimately, the study included 11,892 patients, of whom 3725 received low-intensity LLT, and 8167 received moderate- to high-intensity LLT **(Supplemental Figure 1** and **Supplemental Table 4)**.

Table 1 details the baseline characteristics of the included patients. Compared with those receiving low-intensity LLT, patients on moderate- to high-intensity LLT were slightly younger, had a higher proportion of males, fewer complications such as heart failure, chronic kidney diseases, and atrial fibrillation, and were more likely to have a prior statin prescription within six months before admission. And those on moderate- to high-intensity LLT had higher in-hospital usage rates of DAPT, ACEI/ARB, and *β*-blockers, as well as a higher proportion undergoing PCI.

**Table 1:** Baseline characteristics according to lipid-lowering therapy.

Variables	Total cohort *n* = 11,892	Low-intensity *n* = 3725	Moderate- to High-intensity *n* = 8167	*P* value
Age (year)	83.0 (81.0, 86.0)	84.0 (81.0, 87.0)	83.0 (81.0, 86.0)	<0.001
Male, n ( %)	5840 (49.1)	1745 (46.8)	4095 (50.1)	<0.001
Married, n ( %)	8261 (69.5)	2566 (68.9)	5695 (69.7)	0.35
Year of Admission	2018 (2016, 2020)	2017 (2015, 2019)	2018 (2016, 2020)	<0.001
STEMI, n ( %)	4554 (38.3)	1196 (32.1)	3358 (41.1)	<0.001
Previous history, n ( %)				
Myocardial infarction	1605 (13.5)	526 (14.1)	1079 (13.2)	0.18
PCI	1088 (9.1)	248 (6.7)	840 (10.3)	<0.001
Diabetes mellitus	3507 (29.5)	1016 (27.3)	2491 (30.5)	<0.001
Hypertension	8209 (69.0)	2535 (68.1)	5674 (69.5)	0.12
Heart failure	6318 (53.1)	2203 (59.1)	4115 (50.4)	<0.001
Chronic kidney disease	1799 (15.1)	615 (16.5)	1184 (14.5)	0.004
Atrial fibrillation	2436 (20.5)	833 (22.4)	1603 (19.6)	<0.001
Stroke	1070 (9.0)	353 (9.5)	717 (8.8)	0.22
Chronic obstructive pulmonary disease	1507 (12.7)	574 (15.4)	933 (11.4)	<0.001
Cancer	297 (2.5)	95 (2.6)	202 (2.5)	0.80
Peripheral arterial disease	1818 (15.3)	487 (13.1)	1331 (16.3)	<0.001
CCI score	2 (0, 5)	2 (0, 4)	2 (0, 5)	<0.001
Laboratory test				
Total cholesterol, mg/dL	168.0 (141.1, 198.0)	168.2 (141.5, 197.2)	167.8 (140.8, 198.4)	0.61
LDL-C, mg/dL	106.0 (83.5, 131.1)	104.4 (82.8, 128.4)	106.3 (83.9, 132.3)	0.002
HDL-C, mg/dL	42.2 (35.2, 50.3)	42.9 (35.6, 51.8)	41.8 (35.2, 49.5)	<0.001
Triglycerides, mg/dL	100.1 (74.4, 134.6)	97.4 (71.7, 130.2)	101.0 (76.2, 137.3)	<0.001
eGFR, mL/min/1.73m^2^	59.4 (43.1, 76.3)	57.6 (41.3, 74.3)	60.2 (44.0, 76.9)	<0.001
Haemoglobin, g/L	120.0 (107.0, 132.0)	119.0 (106.0, 131.3)	120.0 (107.0, 132.0)	0.098
Platelet, 10^9^/L	203.0 (168.0, 243.1)	203.0 (167.0, 244.0)	203.0 (169.0, 243.0)	0.96
Pre-hospital medication in the past 6 months, n ( %)				
Antiplatelet	2988 (25.1)	828 (22.2)	2160 (26.4)	<0.001
Statin	2400 (20.2)	693 (18.6)	1707 (20.9)	0.004
*β*-blocker	1519 (12.8)	432 (11.6)	1087 (13.3)	0.009
ACEI/ARB	1908 (16.0)	569 (15.3)	1339 (16.4)	0.12
Calcium channel blockers	1919 (16.1)	532 (14.3)	1387 (17.0)	<0.001
Diuretic	2320 (19.5)	757 (20.3)	1563 (19.1)	0.13
Antidiabetic drug	1999 (16.8)	606 (16.3)	1393 (17.1)	0.29
In-hospital treatment, n ( %)				
DAPT	8907 (74.9)	2396 (64.3)	6511 (79.7)	<0.001
Anticoagulation therapy	9758 (82.1)	2776 (74.5)	6982 (85.5)	<0.001
ACEI/ARB	7533 (63.3)	2179 (58.5)	5354 (65.6)	<0.001
*β*-blockers	7752 (65.2)	2216 (59.5)	5536 (67.8)	<0.001
Calcium channel blockers	3276 (27.5)	1055 (28.3)	2221 (27.2)	0.20
Diuretic	9375 (78.8)	3112 (83.5)	6263 (76.7)	<0.001
Antidiabetic drug	6114 (51.4)	2031 (54.5)	4083 (50.0)	<0.001
PPI	10,862 (91.3)	3240 (87.0)	7622 (93.3)	<0.001
PCI	3516 (29.6)	659 (17.7)	2857 (35.0)	<0.001

**Abbreviation:** ACEI, angiotensin-converting enzyme inhibitor; ARB, angiotensin II receptor blocker; CCI, Charlson Comorbidity Index; DAPT, dual antiplatelet therapy; eGFR, estimated glomerular filtration rate; HDL-C, high-density lipoprotein cholesterol; LDL-C, low-density lipoprotein cholesterol; PCI, percutaneous coronary intervention; PPI, proton pump inhibitor; STEMI, ST-elevation myocardial infarction.

### 3.2 Management and following-up LDL-C level

The baseline levels and changes in LDL-C across four time periods for both groups are illustrated in **Supplemental Figure 2**. The median baseline LDL-C levels for low-intensity and moderate- to high-intensity LLT groups were 104.4 mg/dL and 106.3 mg/dL, respectively. By 30–90 days after admission, LDL-C levels decreased to 87.4 mg/dL in the low-intensity group and 76.6 mg/dL in the moderate- to high-intensity group. Overall, the reduction in LDL-C levels was more pronounced in the moderate- to high-intensity LLT group.

### 3.3 Long-term primary and secondary outcomes

**Fig. 1:**
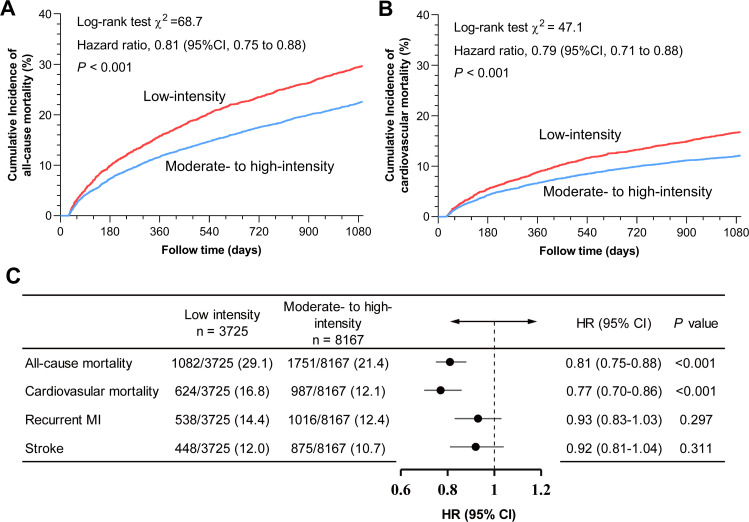
Cumulative incidence of the mortality risk and effects of lipid-lowering therapy on outcomes among patients over 80 years old with myocardial infarction. Panel A: Cumulative all-cause mortality curves over a 3-year follow-up; Panel B: Cumulative cardiovascular mortality curves over a 3-year follow-up; Panel C: Effects of lipid-lowering therapy on primary and secondary outcomes. Red and blue lines represent low-intensity lipid-lowering therapy and moderate- to high-intensity lipid-lowering therapy, respectively. The HR and 95 % CI are visually represented by point estimates and horizontal lines, indicating outcomes favor low-intensity lipid-lowering therapy or moderate- to high-intensity lipid-lowering therapy, with statistical significance noted by *P* values. Statistical significance was tested with multivariable adjusted Cox model and noted by *P* values. Abbreviation: CI, confidence intervals; HR, hazard ratios; MI, myocardial infarction.

During a median follow-up of 3 years, all-cause mortality rates were 29.1 % for patients receiving low-intensity LLT and 21.4 % for those receiving moderate- to high-intensity LLT. Compared with low-intensity LLT group, moderate- to high-intensity LLT was associated with a statistically significant reduction in all-cause mortality at 3-year follow-up (HR, 0.81 [95 % CI: 0.75–0.88]; *P* < 0.001) (Fig. 1**A**). Similar results were observed in the Cox regression analysis after PSM (HR, 0.80 [95 % CI: 0.73–0.88]; *P* = 0.001) (**Supplemental Figure 3**). The variables in the PSM cohort were well balanced between the two groups (**Supplemental Table 5** and **Supplemental Figure 4**).

Compared to low-intensity LLT, moderate- to high-intensity LLT also demonstrated a reduced three-year incidence of cardiovascular mortality (HR, 0.77 [95 % CI: 0.70–0.86]; *P* < 0.001) (Fig. 1**B**) in the Cox regression analysis. However, during 3 years of follow-up, after the first MI, higher LLT was not associated with the reduction of recurrent MI (HR, 0.93 [95 % CI: 0.83–1.03]; *P* = 0.170) or stroke (HR, 0.92 [95 % CI: 0.81–1.04]; *P* = 0.169) (Fig. 1**C**).

### 3.4 Subgroup analysis

**Fig. 2:**
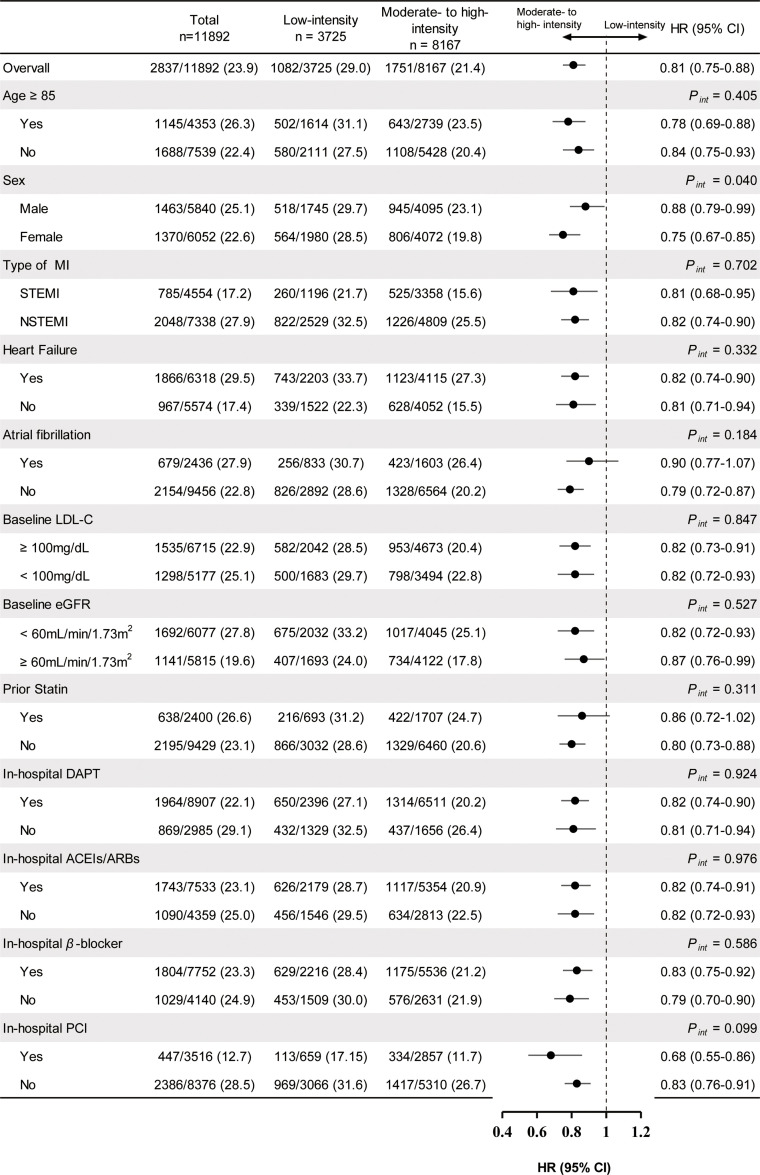
Subgroup analysis of 3-year all-cause mortality in patients over 80 years old with myocardial infarction. The HR and 95 % CI are visually represented by point estimates and horizontal lines, indicating outcomes favor low-intensity lipid-lowering therapy or moderate- to high-intensity lipid-lowering therapy, with statistical significance noted by P values. Abbreviation: ACEI, angiotensin converting enzyme inhibitors; ARB, angiotensin receptor blockers; CI, confidence intervals; DAPT, dual antiplatelet therapy; eGFR, estimated glomerular filtration rate; HR, hazard ratios; LDL-C, low-density lipoprotein cholesterol; MI, myocardial infarction; NSTEMI, non-ST-elevation myocardial infarction; PCI, percutaneous coronary intervention; STEMI, ST-elevation myocardial infarction.

In subgroup analyses, female patients appeared to benefit more from higher intensity LLT (HR, 0.75 [95 % CI: 0.67–0.85]) than male patients (HR, 0.88 [95 % CI: 0.79–0.99]), with a statistically significant interaction (*P_int_* = 0.040). The interaction *P* values were non-significant in other prespecified subgroup analyses (Fig. 2).

### 3.5 Interactions between CCI and LLT intensity on mortality

**Fig. 3:**
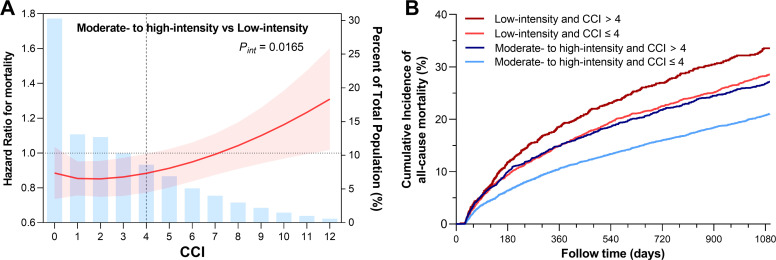
Interactions between Charlson Comorbidity Index and lipid-lowering therapy intensity on all-cause mortality Panel A: MFPI analysis of the effect of LLT intensity on mortality across different levels of CCI. Panel B: Cumulative all-cause mortality risk stratified by the intensity of LLT stratified into four groups based on CCI scores (≤ 4 vs. > 4) and LLT intensity (low vs. moderate- to high-). Abbreviation: CCI, Charlson Comorbidity Index.

**Fig. 4:**
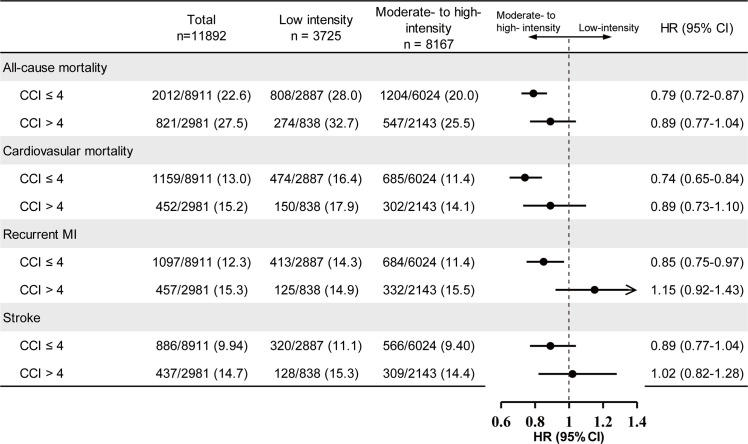
Effects of lipid-lowering therapy on primary and secondary outcomes stratified by CCI scores The HR and 95 % CI are visually represented by point estimates and horizontal lines, indicating outcomes favor low-intensity lipid-lowering therapy or moderate- to high-intensity lipid-lowering therapy. Statistical significance was tested with multivariable adjusted Cox model. Abbreviation: CCI, Charlson Comorbidity Index; CI, confidence intervals; HR, hazard ratios; MI, myocardial infarction.

The MFPI analysis revealed a significant interaction between the CCI and the intensity of LLT on all-cause mortality (interaction *P* = 0.017). As the CCI increased, the mortality risk reduction associated with moderate- to high-intensity LLT, compared to low-intensity LLT, progressively diminished. (Fig. 3**A**) Patients with CCI scores ≤ 4 demonstrated the greatest reduction in both all-cause mortality and cardiovascular mortality when treated with moderate- to high-intensity LLT (HR, 0.79 [95 % CI: 0.72–0.87], *P* < 0.001; HR, 0.74 [95 % CI: 0.65–0.84], *P* < 0.001, respectively). In contrast, no significant reduction in all-cause mortality or cardiovascular mortality was observed with moderate- to high-intensity LLT in patients with CCI scores > 4 (HR, 0.89 [95 % CI: 0.77–1.04], *P* = 0.146; HR, 0.89 [95 % CI: 0.73–1.10], *P* = 0.288, respectively).(Fig. 4). Fig. 3**B** and **Supplemental Figure 5** demonstrates the cumulative incidence of all-cause mortality and cardiovascular mortality for the cohort, stratified into four groups based on CCI scores (≤ 4 vs. >4) and LLT intensity (low vs. moderate- to high-), respectively.

Meanwhile, stratified analyses revealed that moderate-to-high-intensity LLT significantly reduced recurrent MI risk compared to low-intensity LLT in patients with CCI scores ≤ 4 (HR, 0.85 [95 % CI: 0.75–0.97], *P* = 0.018), whereas no benefit was observed in those with CCI > 5 (HR, 1.15 [95 % CI: 0.92–1.43], *P* = 0.222). Notably, stroke incidence remained comparable across LLT intensity groups regardless of CCI stratification (CCI ≤ 4: HR, 0.89 [95 % CI: 0.77–1.04], *P* = 0.139; CCI > 5: HR, 1.02 [95 % CI: 0.82–1.28], *P* = 0.854) (Fig. 4).

In sensitivity analyses, we retained 3637 patients with missing baseline LDL-C and 15,529 MI patients aged over 80 years were ultimately included among a total of 23,478 patients. The results remained consistent in showing that only patients with a CCI score ≤ 4 derived benefit from moderate-to-high intensity LLT compared to low-intensity LLT. **(Supplemental Figure 6)** Furthermore, the sensitivity analyses accounting for competing risk confirmed the robustness of our study findings as well. **(Supplemental Figure 7)**

## 4 Discussion

In this retrospective cohort study based on province-wide electronic health records involving over 10,000 patients, we found that among MI patients aged over 80, moderate- to high-intensity LLT was significantly associated with reduced 3-year all-cause and cardiovascular mortality. Moreover, the CCI score demonstrated a modifying effect on this association, with patients who had lower CCI scores deriving greater benefit from more intensive LLT. These findings complement the current limited evidence on intensive lipid-lowering strategies in very elderly MI patients and provide empirical support and clinical guidance for developing individualized LLT regimens based on comorbidity burden in this high-risk population.

Statin-based LLT is a critical approach to controlling plasma LDL-C levels and reducing adverse cardiovascular events in post-MI patients [[3], [4], [5], [6], [7]]. However, there is limited evidence regarding the optimal intensity of LLT for elderly patients following MI [27,28]. A study by Choudhry and coworkers conducted from 1997 to 2004 [25], which employed a similar grouping method, concluded that elderly patients did not benefit from higher intensity LLT after MI. Nevertheless, with the development and diversification of treatment strategies, the prognosis for this patient population has significantly improved, making our findings unsurprising [29]. In addition, a prior cohort study by Fayol and colleagues in France [28], involving 2258 MI patients aged over 80, demonstrated that high-intensity LLT significantly reduced 5-year mortality risk. Unlike this study conducted in a French population (with an average life expectancy of 82.5 years), our research was carried out in China, a developing country with a shorter average life expectancy (77.4 years).

Notably, the prevalence of multimorbidity increased substantially with age, [30] and prior studies have proved that comorbidities are often associated with worsened short-term clinical outcomes in patients following MI [[31], [32], [33]]. The optimal intensity of LLT in patients with different comorbidity burden remains contentious due to limited evidence from prior studies, particularly given the time-dependent nature of atherosclerotic risk reduction that requires survival horizons exceeding therapeutic latency periods [34,35]. Moreover, concomitant polypharmacy in patients with high comorbidity burden elevates the risk of drug-drug interactions and adverse drug reactions during LLT [36,37]. These factors necessitate personalized LLT regimens tailored to residual lifespan projections and geriatric risk profiles in elderly patients post MI.

Consequently, we calculated a comorbidity score for each patient using CCI, and stratified the cohort according to the scores, revealing that patients with CCI scores ≤ 4 derived significantly both all-cause mortality and cardiovascular mortality reduction from moderate-to-high-intensity LLT, whereas no statistically meaningful benefit was observed in patients with CCI scores > 4. There are several possible explanations for this finding. First, the observed mortality reduction with moderate-to-high-intensity LLT in patients with CCI scores ≤ 4 is likely attributable to their relatively stable conditions, better tolerance to LLT, and fewer complications, making the benefits of LLT more evident [27]. In contrast, patients with severe comorbidities often have complex underlying conditions and multiple complications, such as advanced renal impairment and severe liver disease, which may limit the efficacy of LLT. Patients with multiple comorbidities are more likely to experience adverse events and intolerance to moderate-to-high-intensity LLT, [38] and have competing risks of non-cardiovascular death that limit the benefits of moderate- to high-intensity LLT [39]. This interaction likely explains why moderate-to-high-intensity lipid-lowering therapy reduced recurrent MI risk only in patients with CCI scores ≤ 4, whereas no benefit was observed in unstratified analyses or those with CCI > 4. Therefore, the management of patients with multimorbidity necessitates a holistic evaluation of global health status, projected life expectancy, and susceptibility to treatment-related adverse events to inform personalized therapeutic regimens.

Besides, among MI patients aged over 80, the risk-benefit assessment of LLT requires particularly careful consideration as well. Current evidences and guidelines advocate intensive LLT for secondary prevention to reduce future cardiovascular events [40]. However, while such therapy decrease non-fatal MI, it appears not to reduce all-cause mortality [41], and may potentially increase risks of diabetes mellitus and bleeding complications [42,43]. Furthermore, the critical question remains whether these elderly patients have sufficient life expectancy to derive meaningful clinical benefit from intensive treatment [34,43]. Additional concerns specific to geriatric populations include statin-associated myalgia, myopathy and potential drug-drug interactions [13]. To address these complexities, we employed CCI stratification to identify patient subgroups likely to derive net benefit from moderate-to-high intensity LLT. Additionally, although our cohort contained a limited proportion of patients receiving combination therapy of ezetimibe, PCSK9 inhibitor and statins, published evidence confirms that such medication regimens confer greater therapeutic efficacy and reduced adverse events compared to high-dose statin monotherapy [44,45]. These findings indicate that combination therapy could represent a better option for elderly patients presenting with MI. Further research is warranted to delineate optimized LLT strategies in this population, with the dual objectives of improving treatment efficacy while minimizing adverse effects.

Our study has several limitations. First, it is a retrospective observational study. Although we applied rigorous statistical methods and assessed for residual confounding factors, the potential impact of unmeasured confounding may still exist. There is also clinical decision bias in medication use, as physicians make treatment decisions based on patient-specific conditions [46]. Second, we only recorded in-hospital prescriptions, but in reality, the lipid-lowering medications and dosages for many patients were not constant over time. Adherence to medication regimens is unclear, which could introduce bias into the long-term outcomes. Third, the proportion of patients receiving combination therapy was relatively low in our cohort. Future research is needed to further evaluate the efficacy of combination LLT in elderly patients with MI. Fourth, although our analysis demonstrated benefit in patients with CCI scores ≤ 4, we were unable to incorporate data on myalgia, myopathy, new-onset diabetes, or bleeding events due to challenges in obtaining accurate real-world data on these outcomes. Additionally, this study included only patients from China, so caution is needed when generalizing the results. Further studies in broader populations are necessary to validate our findings.

## 5 Conclusion

In this observational study of MI patients aged 80 years and older, moderate-to-high-intensity LLT significantly reduced all-cause and cardiovascular mortality during 3-year follow-up compared to low-intensity LLT in patients with CCI scores ≤ 4, whereas no mortality difference was observed between LLT intensity groups in those with CCI > 4. The results underscore that an individualized approach to lipid management based on comorbidity status may help optimize cardiovascular protection in very elderly MI patients. Further investigation is required to optimize personalized lipid management through rigorous assessment of LLT benefits versus risks, including adverse effects, diabetes progression, and bleeding events.

## Ethics approval and consent to participate

This study was approved by the Ethics Committee of the Tianjin Medical University General Hospital (IRB2024-YX-131–01). Informed consent was waived due to the retrospective nature of the study.

## Consent for publication

All contributing authors have reviewed and consented to the submission of this manuscript.

## Funding

National Key Research and Development Program of China (No. 2023YFC3605200 and No. 2025YFE0126700) and National Natural Science Foundation of China (82321001, 72274133 and 72304205).

## CRediT authorship contribution statement

**Shichen Jiang:** Writing – original draft, Visualization, Formal analysis, Data curation. **Linjie Li:** Writing – original draft, Visualization, Formal analysis, Data curation. **Yuyang Miao:** Writing – original draft, Visualization, Formal analysis, Data curation. **Geru Aa:** Writing – review & editing. **Hangkuan Liu:** Writing – review & editing. **Xiaozhi Chen:** Writing – review & editing. **Haonan Sun:** Writing – review & editing. **Yiwen Fang:** Writing – review & editing. **Pengfei Sun:** Supervision, Funding acquisition, Conceptualization. **Xin Zhou:** Supervision, Funding acquisition, Conceptualization. **Qiang Zhang:** Supervision, Funding acquisition, Conceptualization.

## Declaration of competing interest

The authors declare that they have no known competing financial interests or personal relationships that could have appeared to influence the work reported in this paper.

## Appendix Supplementary materials

Image, application 1
