# Supplementary material for: Low-intensity versus moderate- to high-intensity lipid-lowering therapy after myocardial infarction in patients aged 80 years and older: A retrospective cohort study
Source: Am J Prev Cardiol. 2025 Dec 21;25:101391. doi: 10.1016/j.ajpc.2025.101391 (PMC12849021; doi:10.1016/j.ajpc.2025.101391)
Supplement: Supplementary file 1 [file mmc1.docx]

**Sup****plemental Material**

**Low-Intensity versus Moderate- to High-Intensity Lipid-Lowering Therapy After Myocardial Infarction in Patients Aged 80 Years and Older: A Retrospective Cohort Study**

By Shichen Jiang, *et al*.

**Table of Contents**

**Data sources**

**Table S1.** Definition of outcomes and comorbidities

**Table S2.** Definition of study variables

**Table S3.** Proportion of missing baseline variables before interpolating

**Table S4.** The frequency of different LLT treatment protocols in AMI patients aged over 80 years

**Table S5.** Baseline characteristics of AMI patients aged over 80 years after propensity score matching

**Figure S1.** Flow diagram of patients including and excluding

**Figure S2.** Serum levels of LDL-C in AMI patients aged over 80 years at baseline and following-up

**Figure S3.** Kaplan-Meier curves of all-cause mortality in AMI patients aged over 80 years after propensity score matching

**Figure S4.** Love plot for absolute standardized differences comparing baseline characteristics between patients receiving low-intensity LLT and moderate- to high-intensity LLT before and after propensity score matching

**Figure S5.** Cumulative cardiovascular mortality risk stratified into four groups based on CCI scores (≤ 4 vs. > 4) and LLT intensity (low- vs. moderate- to high-)

**Figure S6.** Sensitivity analyses in patients over 80 years old with myocardial infarction including missing baseline LDL-C cases

**Figure S7.** Sensitivity analyses accounting for competing risk in patients over 80 years old with myocardial infarction

**Data sources**

The data for this study were obtained from an integrated repository encompassing comprehensive healthcare records from multiple medical centers in China. This comprehensive system incorporates full participation from all tertiary general hospitals (42/42), 97.8% of community healthcare facilities (261/267), and 66.7% of secondary hospitals (36/54), collating clinical data from January 1, 2013, to December 31, 2022. The platform aggregates three primary categories of health data: inpatient records, outpatient encounters, and mortality statistics from community healthcare institutions. The platform's standardized data architecture includes six key components: 1) Demographic characteristics (age, gender, marital status); 2) Clinical diagnoses codified using ICD-10 standards through China's National Clinical Edition 2 codebook; 3) Pharmaceutical prescriptions; 4) Laboratory test outcomes; 5) Procedural intervention codes; and 6) Clinical endpoints encompassing mortality, discharge disposition, and hospitalization duration. All patient records within the system are assigned unique encrypted identifiers enabling longitudinal tracking across multiple healthcare encounters while ensuring compliance with data protection regulations through automated de-identification protocols. This investigation received formal authorization from local health commission and was granted ethical approval by the institutional review board of authors’ affiliation (IRB2024-YX-131-01). The requirement for informed consent was waived pursuant to the retrospective observational design.

**Supplementary Tables and Figures**

**Table S1. Definition of outcomes and comorbidities**

| Outcomes | ICD codes |
| --- | --- |
| Recurrent MI | I21, I22 |
| Stroke | I60, I61, I63 |
| Comorbid conditions | ICD codes |
| MI | I21, I22 |
| ST-Elevation MI | I21.0, I21.1, I21.2, I21.300, I21.300x004, I21.300x011, I22.0, I22.1, I22.8 |
| Diabetes mellitus | E10 - E14, O24 |
| Hypertension | I10 - I15, O10 - O16 |
| Heart failure | I11.0, I13.0, I13.2, I50.0, I50.1, I50.900, I50.900x001, I50.900x002, I50.900x008, I50.900x009, I50.900x010, I50.900x014, I50.900x015, I50.900x016, I50.900x017, I50.900x018, I50.900x019, I50.900x021, I50.900x022, I50.900x023, I50.904, I50.905, I50.906, I50.907, I50.908, J81.x00, J81.x00x002, J95.811, O99.500x008, O99.507, R57.000, I97.000x011, I97.001, I97.100x004, I97.101, I97.102, I97.803, O29.100x002, O29.100x012, O75.403, O89.100x002, O99.400x008, T81.800x01 |
| Chronic kidney disease | E10.200, E11.200, E12.200, E13.200, E14.200, E85.002, I12, I13, I15.0, I15.1, I70.101, K76.7, M35.102, N00.900x007, N00.900x009, N02 - N07, N08.0, N08.1, N08.2, N08.3, N08.4, N08.5, N08.8, N14, N15, N16.0, N16.1, N16.2, N16.3, N16.4, N16.5, N16.8, N18, N19, N25 - N27, N28.0, N28.9, N29.8, N29.1 N99.0, P96.0, Q27.1, Q27.2, Q27.305, Q27.806, Q60 - Q63, R39.2, Z49, Z94.0, Z99.2 |
| Atrial fibrillation | I48 |
| Stroke | I60, I61, I63, I64, G45 |
| Chronic obstructive pulmonary disease | J42, J44, J41.0, J43.1, J43.2, J43.8, J43.9, J20.900x004 |
| Cancer | C00 – C41, C43 – C58, C60 – C85, C88, C90 - C97 |
| Peripheral arterial disease | I70 - I74 |

**Abbreviations**: ICD, international classification of diseases; MI, myocardial infarction;

**Table S2. Definition of study variables**

| **Variables** | **Definition** | **Continuous/Categorical/Binary** |
| --- | --- | --- |
| *Patient demographics* | | |
| Age | Years after birth | Continuous, per 1 year |
| Sex | Female/Male | Binary, Female/Male |
| Marital status | Marital status at enrollment (Married or Unmarried) | Binary, Married/Unmarried |
| *Comorbid Conditions* |  |  |
| Old myocardial infarction | Previous history of myocardial infarction | Binary, Yes/No |
| Previous PCI | Previous PCI treatment | Binary, Yes/No |
| Diabetes mellitus | Currently diagnosed with diabetes mellitus at discharge, regardless of duration of disease or need for antidiabetic agents | Binary, Yes/No |
| Hypertension | Currently diagnosed with hypertension at discharge | Binary, Yes/No |
| Heart failure | Currently diagnosed with heart failure at discharge | Binary, Yes/No |
| Chronic kidney disease | Currently diagnosed with chronic kidney disease at discharge | Binary, Yes/No |
| Atrial fibrillation | Currently diagnosed with atrial fibrillation at discharge | Binary, Yes/No |
| Stroke | Currently diagnosed with stroke at discharge | Binary, Yes/No |
| Chronic obstructive pulmonary disease | Currently diagnosed with chronic obstructive pulmonary disease at discharge | Binary, Yes/No |
| Cancer | Currently diagnosed with cancer at discharge | Binary, Yes/No |
| Peripheral arterial disease | Currently diagnosed with peripheral arterial disease at discharge | Binary, Yes/No |
| *Laboratory test results* |  |  |
| Total cholesterol | First TC measurement after admission | Continuous, per 1.0 mmol/L |
| LDL-C | First LDL-C measurement after admission | Continuous, per 1.0 mmol/L |
| HDL-C | First HDL-C measurement after admission | Continuous, per 1.0 mmol/L |
| Triglycerides | First triglycerides measurement after admission | Continuous, per 1.0 mmol/L |
| eGFR | Calculated using CKD-EPI equation | Continuous, per 1.0 mL/min/1.73m^2^ |
| Haemoglobin | First haemoglobin measurement after admission | Continuous, per 1.0 g/dL |
| Platelet | First platelet measurement after admission | Continuous, per 1.0*10^9^/L |
| *Pre-hospital medications* |  |  |
| Antiplatelet | At least one antiplatelet medication prescription within 183 days prior to enrollment | Binary, Yes/No |
| Statin | At least one statin medication prescription within 183 days prior to enrollment | Binary, Yes/No |
| *β*-blockers | At least one *β*-blockers medication prescription within 183 days prior to enrollment | Binary, Yes/No |
| ACEI/ARB | At least one ACEI/ARB medication prescription within 183 days prior to enrollment | Binary, Yes/No |
| CCB | At least one CCB medication prescription within 183 days prior to enrollment | Binary, Yes/No |
| Diuretic | At least one Diuretic medication prescription within 183 days prior to enrollment | Binary, Yes/No |
|  |  |  |
| *In-hospital management* |  |  |
| DAPT | DAPT status within 24 h of first medical contact: (i) DAPT intensity: non-loading DAPT (DAPT was not in loading dose), single-loading DAPT (one of DAPT in loading dose), and both-loading DAPT (DAPT both in loading dose); (ii) P2Y12 inhibitor used: ticagrelor vs. clopidogrel | Binary, Yes/No |
| Oral anticoagulants | At least one oral anticoagulant prescription during hospitalization | Binary, Yes/No |
| ACEI/ARB | At least one ACEI/ARB prescription during hospitalization | Binary, Yes/No |
| *β*-blockers | At least one *β*-blocker prescription during hospitalization | Binary, Yes/No |
| Calcium channel blockers | At least one CCB prescription during hospitalization | Binary, Yes/No |
| Diuretic agents | At least one diuretic prescription (oral or intravenous) during hospitalization | Binary, Yes/No |
| Antidiabetic agents | At least one antidiabetic prescription during hospitalization | Binary, Yes/No |
| PPI | At least one PPI prescription during hospitalization | Binary, Yes/No |
| PCI | PCI treatment during hospitalization | Binary, Yes/No |

**Abbreviations**: ACEI, angiotensin-converting enzyme inhibitor; ARB, angiotensin II receptor blocker; DAPT, dual antiplatelet therapy; eGFR, estimated glomerular filtration rate; HDL-C, high-density lipoprotein cholesterol; LDL-C, low-density lipoprotein cholesterol; PCI, percutaneous coronary intervention; PPI, proton pump inhibitor

**Table S3. Proportion of missing baseline variables before interpolating**

|  | Missing / Total | Percent, % |
| --- | --- | --- |
| Total cholesterol, mmol/L | 203 / 11892 | 1.71 |
| HDL-C, mmol/L | 144 / 11892 | 1.21 |
| Triglycerides, mmol/L | 66 / 11892 | 0.55 |
| eGFR, mL/min/1.73m2 | 1320 / 11892 | 11.1 |
| Haemoglobin, g/dL | 209 / 11892 | 1.76 |
| Platelet, 10^9^/L | 1558 / 11892 | 13.1 |

**Abbreviation**: eGFR, estimated glomerular filtration rate; HDL-C, high-density lipoprotein cholesterol; LDL-C, low-density lipoprotein cholesterol

**Table S4. The frequency of different LLT treatment protocols in AMI patients aged over 80 years**

| Low-intensity  n = 3725 | Moderate- to High-intensity  n = 8167 |
| --- | --- |
| Rosuvastatin 5mg: 850 (22.8%)  Atorvastatin 10mg or 15mg: 1576 (42.3%)  Other statins*: 1297 (34.8%)  Ezetimibe: 2 (0.05%) | Rosuvastatin 10mg: 2711 (33.2%)  Rosuvastatin more than 10mg: 66 (0.81%)  Atorvastatin 20mg: 4710 (57.7%)  Atorvastatin more than 20mg: 262 (3.21%)  Combination of ezetimibe, PCSK9 inhibitor and statins: 418 (5.12%) |

*Other statins include pravastatin, fluvastatin, simvastatin, ‌pitavastatin and lovastatin.

**Abbreviation:** PCSK9, proprotein convertase subtilisin/kexin type 9

**Table S5. Baseline characteristics of AMI patients aged over 80 years after propensity score matching**

| Variables | Total cohort  n = 6832 | Low-intensity  n = 3416 | Moderate- to High-intensity  n = 3416 | ASD (%) |
| --- | --- | --- | --- | --- |
| Age (year) | 84.0 (81.0, 87.0) | 84.0 (81.0, 86.0) | 84.0 (81.0, 87.0) | 0.48 |
| Male, n (%) | 3272 (47.9) | 1617 (47.3) | 1655 (48.4) | 2.23 |
| Married, n (%) | 4729 (69.2) | 2358 (69.0) | 2371 (69.4) | 0.82 |
| Year of Admission | 2017 (2015, 2019) | 2017 (2015, 2019) | 2017 (2015, 2019) | 5.38 |
| STEMI, n (%) | 2260 (33.1) | 1115 (32.6) | 1145 (33.5) | 1.87 |
| Previous history, n (%) | | | | |
| Myocardial infarction | 941 (13.8) | 486 (14.2) | 455 (13.3) | 2.63 |
| PCI | 498 (7.3) | 245 (7.2) | 253 (7.4) | 0.90 |
| Diabetes mellitus | 1931 (28.3) | 964 (28.2) | 967 (28.3) | 0.20 |
| Hypertension | 4684 (68.6) | 2340 (68.5) | 2344 (68.6) | 0.25 |
| Heart failure | 3896 (57.0) | 1980 (58.0) | 1916 (56.1) | 3.79 |
| Chronic kidney diseases | 1114 (16.3) | 565 (16.5) | 549 (16.1) | 1.27 |
| Atrial fibrillation | 1485 (21.7) | 762 (22.3) | 723 (21.2) | 2.77 |
| Stroke | 655 (9.6) | 332 (9.7) | 323 (9.5) | 0.90 |
| Chronic obstructive pulmonary disease | 962 (14.1) | 493 (14.4) | 469 (13.7) | 2.02 |
| Cancer | 180 (2.6) | 89 (2.6) | 91 (2.7) | 0.37 |
| Peripheral arterial disease | 934 (13.7) | 459 (13.4) | 475 (13.9) | 1.36 |
| Laboratory test | | | | |
| Total cholesterol, mg/dL | 168.2 (141.1, 198.0) | 168.2 (141.7, 197.2) | 167.8 (140.4, 198.8) | 0.56 |
| LDL-C, mg/dL | 98.3 (73.5, 132.9) | 98.3 (72.6, 132.0) | 98.3 (74.4, 133.7) | 0.47 |
| HDL, mg/dL | 104.8 (82.0, 129.5) | 105.2 (83.1, 129.2) | 104.4 (81.6, 130.7) | 2.33 |
| Triglycerides, mg/dL | 42.5 (35.6, 51.0) | 42.5 (35.6, 51.4) | 42.5 (35.6, 50.7) | 2.49 |
| eGFR, mL/min/1.73m^2^ | 57.7 (41.6, 74.6) | 57.7 (41.3, 74.8) | 58.0 (41.8, 74.6) | 1.83 |
| Haemoglobin, g/dL | 119.0 (106.0, 131.0) | 119.0 (106.0, 132.0) | 120.0 (106.0, 131.0) | 0.64 |
| Platelet, 10^9^/L | 203.0 (169.0, 243.0) | 203.0 (169.0, 245.0) | 202.1 (169.0, 241.2) | 2.33 |
| Pre-hospital medication in the past 6 months, n (%) | | | | |
| Antiplatelet | 1598 (23.4) | 791 (23.2) | 807 (23.6) | 1.11 |
| Statin | 1316 (19.3) | 649 (19.0) | 667 (19.5) | 1.34 |
| *β*-blockers | 831 (12.2) | 406 (11.9) | 425 (12.4) | 1.70 |
| ACEI/ARB | 1074 (15.7) | 536 (15.7) | 538 (15.7) | 0.16 |
| Calcium channel blockers | 1040 (15.2) | 516 (15.1) | 524 (15.3) | 0.65 |
| Diuretic | 1392 (20.4) | 706 (20.7) | 686 (20.1) | 1.45 |
| Antidiabetic drug | 1151 (16.8) | 574 (16.8) | 577 (16.9) | 0.24 |
| In-hospital treatment, n (%) | | | | |
| DAPT | 4669 (68.3) | 2303 (67.4) | 2366 (69.3) | 3.97 |
| Anticoagulation therapy | 5349 (78.3) | 2630 (77.0) | 2719 (79.6) | 6.32 |
| ACEI/ARB | 4129 (60.4) | 2049 (60.0) | 2080 (60.9) | 1.86 |
| *β*-blocker | 4188 (61.3) | 2079 (60.9) | 2109 (61.7) | 1.80 |
| Calcium channel blockers | 1976 (28.9) | 988 (28.9) | 988 (28.9) | < 0.001 |
| Diuretic | 5587 (81.8) | 2832 (82.9) | 2755 (80.6) | 5.84 |
| Antidiabetic drug | 3639 (53.3) | 1837 (53.8) | 1802 (52.8) | 2.05 |
| PPI | 6100 (89.3) | 3025 (88.6) | 3075 (90.0) | 4.73 |
| PCI | 1407 (20.6) | 655 (19.2) | 752 (22.0) | 7.03 |

**Abbreviation:** ACEI, angiotensin-converting enzyme inhibitor; ARB, angiotensin II receptor blocker; CCI, Charlson Comorbidity Index; DAPT, dual antiplatelet therapy; eGFR, estimated glomerular filtration rate; HDL-C, high-density lipoprotein cholesterol; LDL-C, low-density lipoprotein cholesterol; PCI, percutaneous coronary intervention; PPI, proton pump inhibitor; STEMI, ST-elevation myocardial infarction

**Figure S1. Flow diagram of patients including and excluding**

**
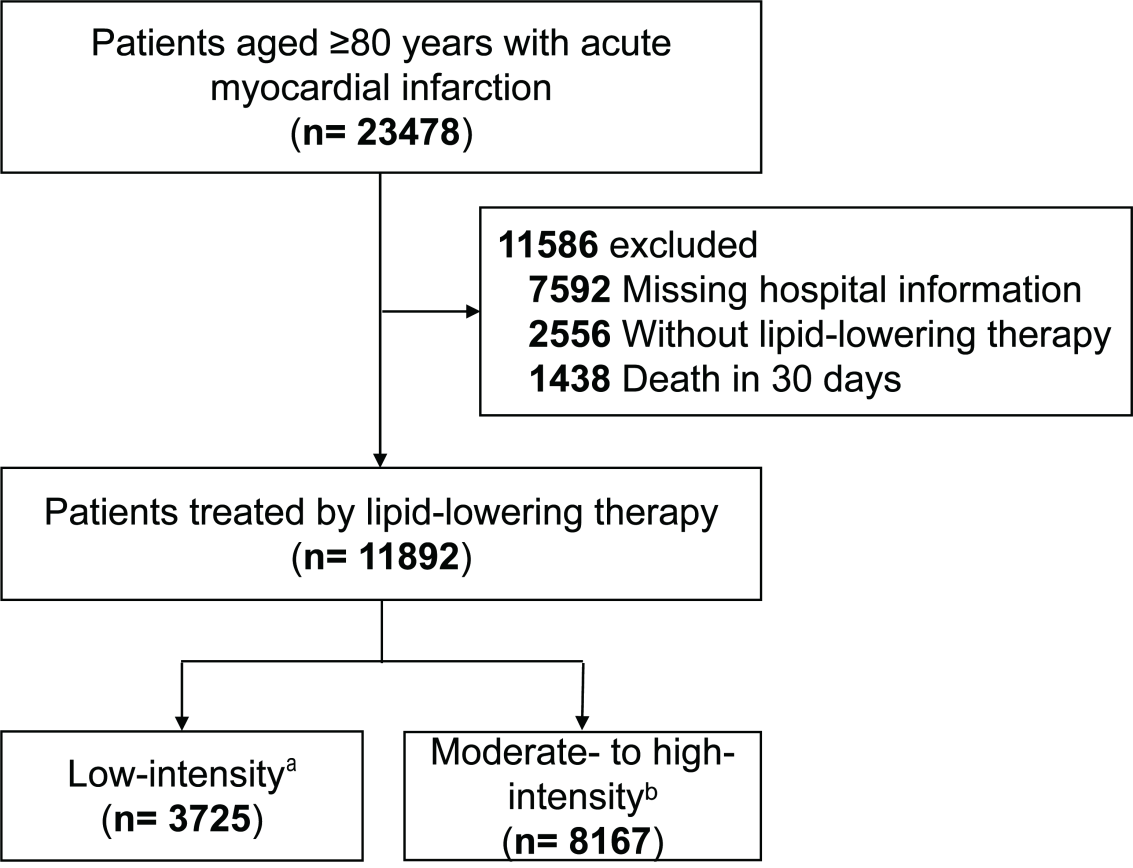
**

^a^ 5mg rosuvastatin, 10mg and 15mg atorvastatin, other statins

^b^ Rosuvastatin ≥10 mg, atorvastatin ≥20 mg, PCSK9 inhibitor, any combination of ezetimibe, PCSK9 inhibitor and statins

**Abbreviation:** PCSK9, proprotein convertase subtilisin/kexin type 9

**Figure S2. Serum levels of LDL-C in AMI patients aged over 80 years at baseline and following-up**


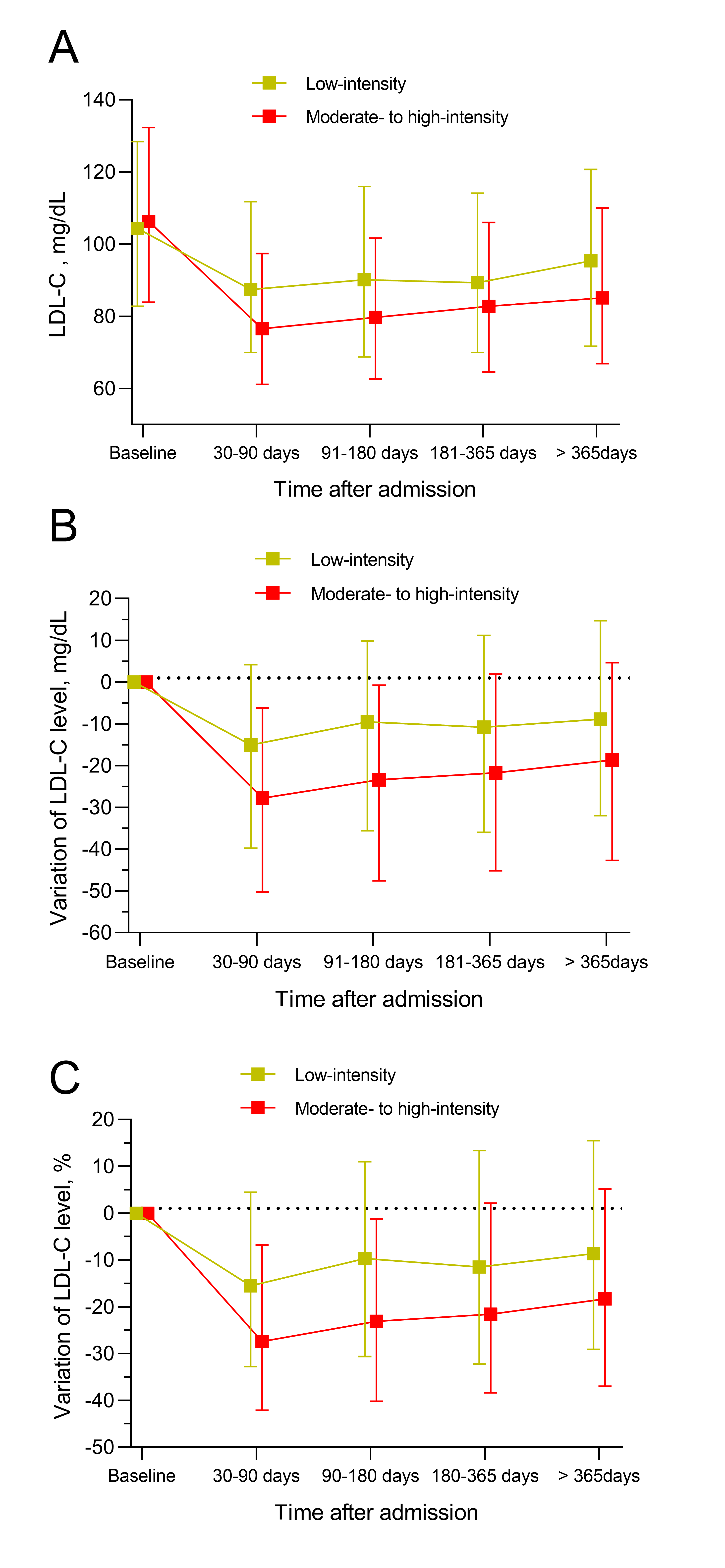


Panel A: The serum levels of LDL-C in patients aged over 80 years at baseline and following-up; Panel B: The absolute change in LDL-C from baseline during follow-up; Panel C: The percent change in LDL-C compared with baseline LDL during follow-up.

The median value and quartitles are visually represented by point estimates and vertical lines.

**Abbreviation**: LDL-C, low-density lipoprotein cholesterol

**Figure S3. Kaplan-Meier curves of all-cause mortality in AMI patients aged over 80 years after propensity score matching**


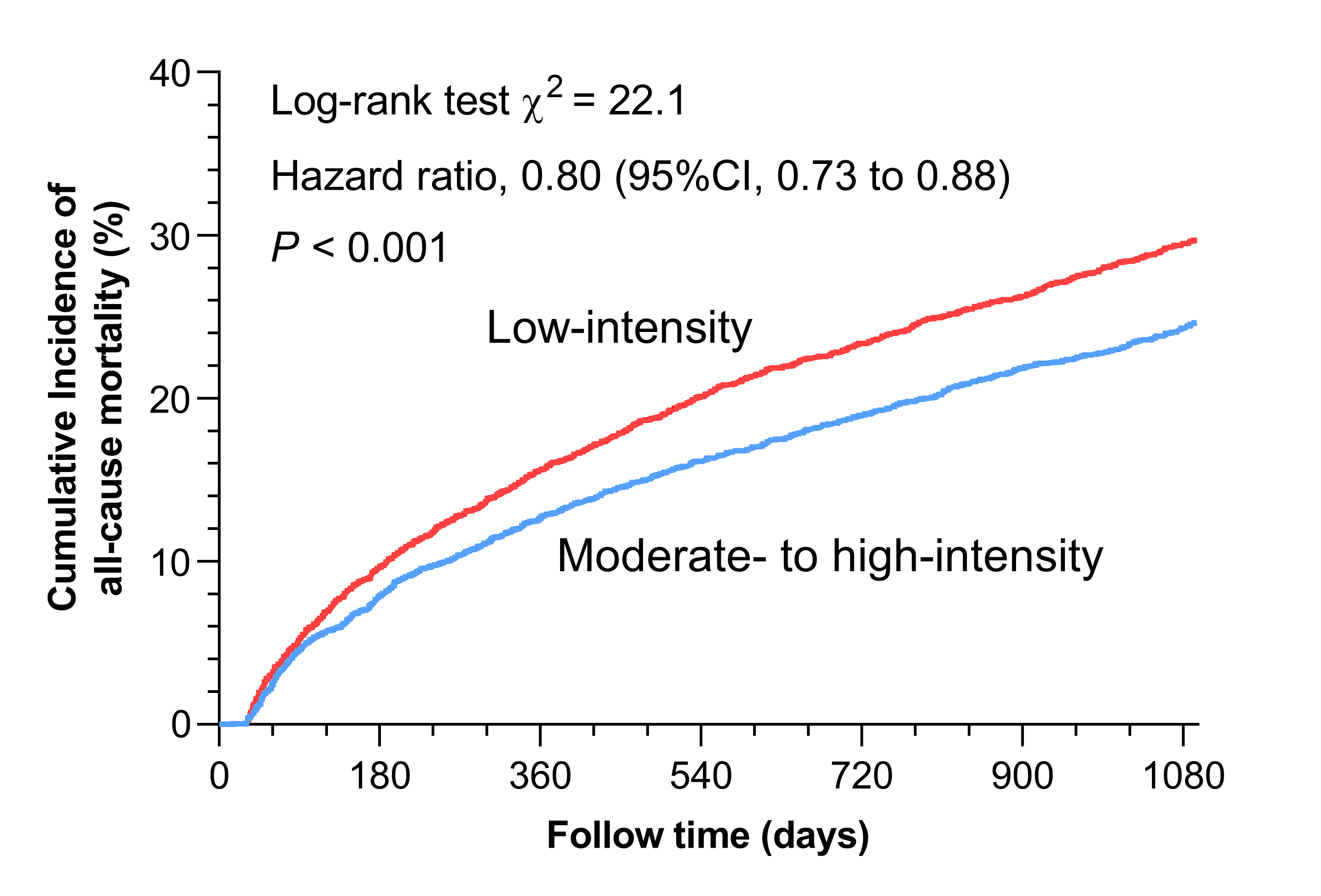


Cox regression models were used to calculate statistical significance noted by P values.

**Figure S4. Love plot for absolute standardized differences comparing baseline characteristics between patients receiving low-intensity LLT and moderate- to high-intensity LLT before and after propensity score matching**


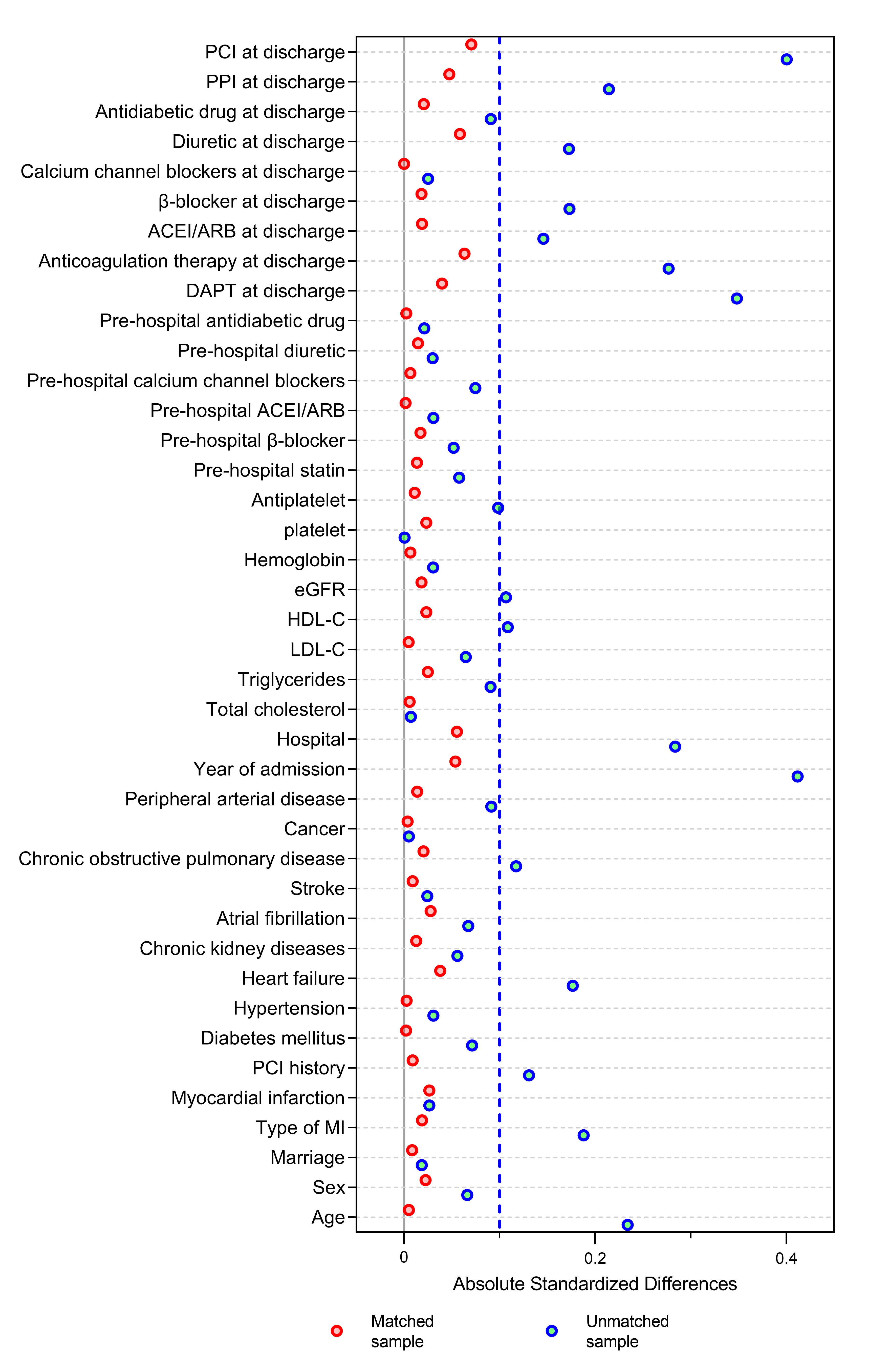


**Abbreviation**:ACEI, angiotensin-converting enzyme inhibitor; ARB, angiotensin II receptor blocker; CCI, Charlson Comorbidity Index; DAPT, dual antiplatelet therapy; eGFR, estimated glomerular filtration rate; HDL-C, high-density lipoprotein cholesterol; LDL-C, low-density lipoprotein cholesterol; PCI, percutaneous coronary intervention; PPI, proton pump inhibitor; STEMI, ST-elevation myocardial infarction

**Figure S5. Cumulative cardiovascular mortality risk stratified into four groups based on CCI scores (≤ 4 vs. > 4) and LLT intensity (low- vs. moderate- to high-)**


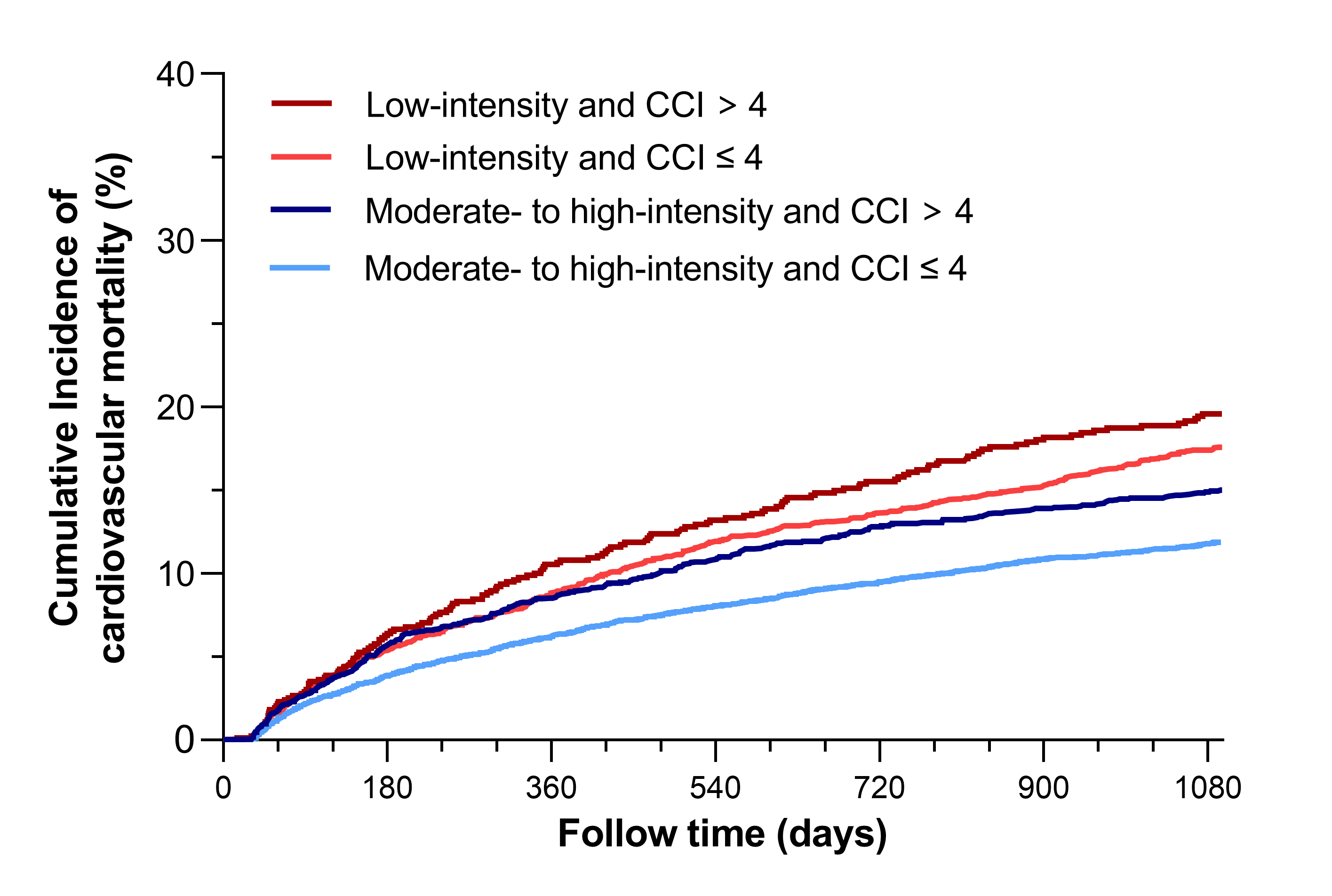


**Abbreviation**: CCI, Charlson Comorbidity Index

**Figure S6. Sensitivity analyses in patients over 80 years old with myocardial infarction including missing baseline LDL-C cases**


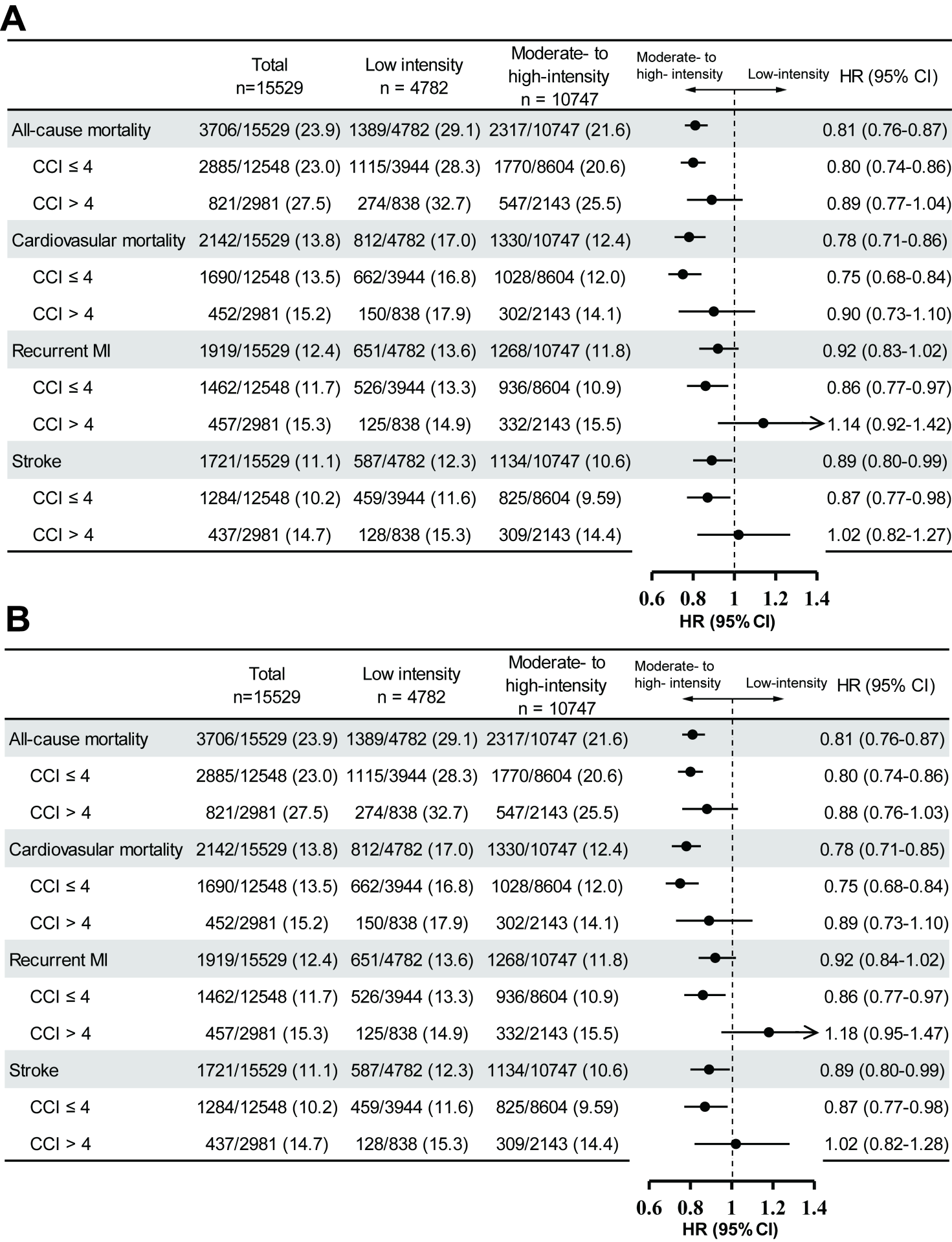


Panel A: Effects of lipid-lowering therapy on outcomes in total cohort and stratified by CCI scores after imputing missing baseline LDL-C values by MissForest; Panel B:Effects of lipid-lowering therapy on outcomes in total cohort and stratified by CCI scores without adjusting for baseline LDL-C.

The HR and 95% CI are visually represented by point estimates and horizontal lines, indicating outcomes favor low-intensity lipid-lowering therapy or moderate- to high-intensity lipid-lowering therapy.

**Abbreviation**: CCI, Charlson Comorbidity Index; CI, confidence intervals; HR, hazard ratios; MI, myocardial infarction

**Figure S7. Sensitivity analyses accounting for competing risk in patients over 80 years old with myocardial infarction**


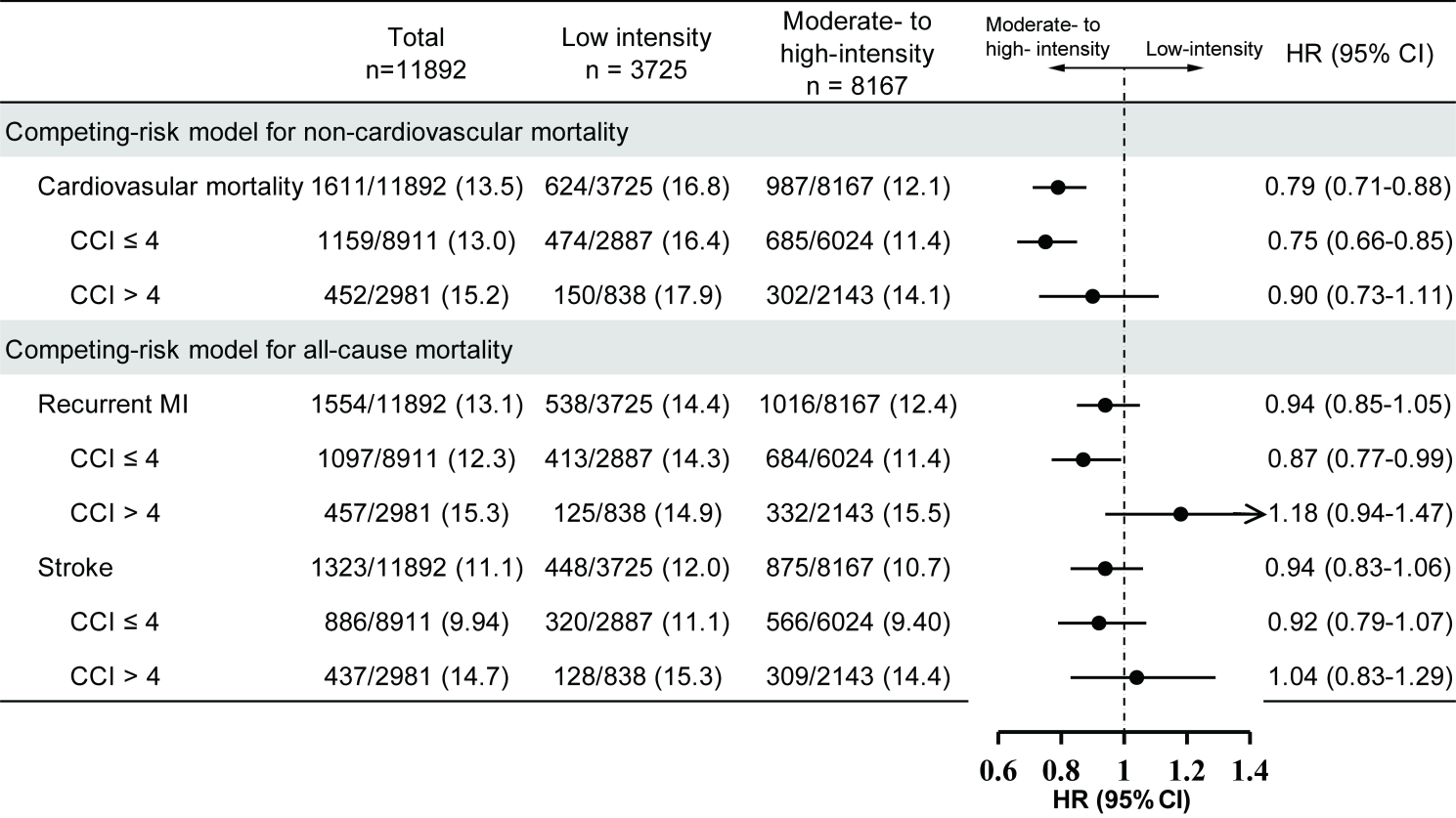


The HR and 95% CI are visually represented by point estimates and horizontal lines, indicating outcomes favor low-intensity lipid-lowering therapy or moderate- to high-intensity lipid-lowering therapy.

**Abbreviation**: CCI, Charlson Comorbidity Index; CI, confidence intervals; HR, hazard ratios; MI, myocardial infarction
